# Evaluation of genomic selection models using whole genome sequence data and functional annotation in Belgian Blue cattle

**DOI:** 10.1186/s12711-025-00955-5

**Published:** 2025-03-04

**Authors:** Can Yuan, Alain Gillon, José Luis Gualdrón Duarte, Haruko Takeda, Wouter Coppieters, Michel Georges, Tom Druet

**Affiliations:** 1 Unit of Animal Genomics, GIGA-R & Faculty of Veterinary Medicine, University of Liège, Avenue de l’Hôpital, 1, 4000 Liège, Belgium; 2 Walloon Breeders Association, Rue Des Champs Elysées, 4, 5590 Ciney, Belgium

## Abstract

**Background:**

The availability of large cohorts of whole-genome sequenced individuals, combined with functional annotation, is expected to provide opportunities to improve the accuracy of genomic selection (GS). However, such benefits have not often been observed in initial applications. The reference population for GS in Belgian Blue Cattle (BBC) continues to grow. Combined with the availability of reference panels of sequenced individuals, it provides an opportunity to evaluate GS models using whole genome sequence (WGS) data and functional annotation.

**Results:**

Here, we used data from 16,508 cows, with phenotypes for five muscular development traits and imputed at the WGS level, in combination with in silico functional annotation and catalogs of putative regulatory variants obtained from experimental data. We evaluated first GS models using the entire WGS data, with or without functional annotation. At this marker density, we were able to run two approaches, assuming either a highly polygenic architecture (GBLUP) or allowing some variants to have larger effects (BayesRR-RC, a Bayesian mixture model), and observed an increased reliability compared to the official GBLUP model at medium marker density (on average 0.016 and 0.018 for GBLUP and BayesRR-RC, respectively). When functional annotation was used, we observed slightly higher reliabilities with an extension of GBLUP that included multiple polygenic terms (one per functional group), while reliabilities decreased with BayesRR-RC. We then used large subsets of variants selected based on functional information or with a linkage disequilibrium (LD) pruning approach, which allowed us to evaluate two additional approaches, BayesCπ and Bayesian Sparse Linear Mixed Model (BSLMM). Reliabilities were higher for these panels than for the WGS data, with the highest accuracies obtained when markers were selected based on functional information. In our setting, BSLMM systematically achieved higher reliabilities than other methods.

**Conclusions:**

GS with large panels of functional variants selected from WGS data allowed a significant increase in reliability compared to the official genomic evaluation approach. However, the benefits of using WGS and functional data remained modest, indicating that there is still room for improvement, for example by further refining the functional annotation in the BBC breed.

**Supplementary Information:**

The online version contains supplementary material available at 10.1186/s12711-025-00955-5.

## Background

The implementation of genomic selection [1] in livestock species has been made possible by the development of high-throughput genotyping technologies. Indeed, the availability of low-cost genotyping arrays has led to the rapid adoption of genomic selection in many livestock species and breeds [2]. However, the availability of whole genome sequence (WGS) should make it possible to further improve the accuracy of genomic selection, as causative variants would be included in the model. Furthermore, with sequence-based genomic selection, the accuracy of predictions would remain high over multiple generations, as the linkage disequilibrium (LD) between markers and causative variants would not decay over generations. With the ability to sequence large reference panels of individuals [3, 4] and the availability of efficient genotype phasing and imputation tools [5–8], it is becoming increasingly common to have imputed WGS data for large cohorts of individuals. However, in early studies based on either simulated or real data, the use of imputed WGS data resulted in no or small improvements in prediction accuracy when the prediction methods were not changed [9–13], whereas predictions using only the causative variants provided a significant improvement [10], especially when they were rare [9].

To fully exploit the potential of whole genome sequence information, other strategies are needed. Two main directions have been proposed in the literature: (1) using additional information to classify variants into different functional categories having different effect sizes; (2) selecting a subset of markers that are more likely to be causative from the whole-genome sequence data, either to reduce model dimensionality or to add the markers to custom genotyping arrays. Two main groups of methods developed to apply the first strategy are commonly used. The first group includes extensions of the genomic best linear unbiased prediction (GBLUP) that fit multiple polygenic terms with their own genomic relationship matrix (GRM), such as the genomic feature BLUP (GFBLUP) [14, 15]. With the GFBLUP, annotation groups are fitted one by one (next to a polygenic term that fits the rest of the genome), but models that fit more than two annotation groups are possible, as in the MultiBLUP model [16]. The second group includes extensions of the BayesR model [17], a Bayesian mixture of Gaussian distributions associated with different SNP effect sizes, including BayesRC [18], BayesRCO [19] and BayesRR-RC [20] models. With both approaches, genetic variants are classified into different annotation groups, which may have group-specific parameters such as effect variances or mixture parameters. The GFBLUP approach has been used to perform heritability partitioning and genomic prediction using different features, such as genome-wide association studies (GWAS) results, expression QTLs (eQTL) [21] and Gene Ontology categories [22]. GFBLUP was found to be more accurate than GBLUP in several studies, although not systematically. McLeod et al. [18] used BayesRC using annotation categories related to coding and putative regulatory variants, specific to lactation genes or not, and achieved slightly higher accuracies compared to a traditional BayesR model. A method similar to BayesRC has also been shown to be efficient for predicting complex traits in humans [20, 23]. However, these annotation-aware approaches have rarely been applied to complete whole-genome sequence data in livestock species (especially the Bayesian approach), and strategies based on marker pre-selection are often implemented. After this marker selection step, genomic predictions can be applied with or without grouping variables according to relevant features. It is common to select markers based on GWAS results [4, 12, 13, 24–26], but other criteria have also been used such as coding variants [13, 26], eQTL [27], putative regulatory regions [26] or more general genomic annotations based on position relative to genes [25]. Xiang et al. [28] used probably the most complete set of criteria in cattle, including functional and evolutionary information, and proposed a global score for each marker. Finally, although most of the time genotypes from selected markers are imputed, there are sometimes included on custom genotyping arrays [26, 29].

The main objective of the present study was to evaluate strategies to improve the accuracy of genomic selection in Belgian Blue cattle (BBC) using imputed whole genome sequence data and functional annotation. This breed is mainly selected for muscular development traits, with the fixation of an 11-bp deletion in the myostatin (*MSTN*) gene associated with double muscling. Recent studies have improved our knowledge of the genetic architecture of these traits. First, selective sweeps revealed that large effect variants have been fixed by selection [30, 31], but only two of the identified hard sweeps were associated with complex traits, and only one was breed-specific and related to muscularity (the *MSTN* mutation). This is consistent with the review by Kemper and Goddard [32], who stated that most loci associated with complex traits in cattle have small effects, but that variants with larger effects can occasionally segregate in the population. Next, a recent sequence-based GWAS study [33] showed that the significant associations are enriched for common coding variants with large effects. However, these correspond to a relatively small number of variants (< 15), those with the largest effects, and contribute only to a small proportion of the genetic variance. In line with this, Yuan et al. [34] estimated that putative regulatory variants have the highest contribution to heritability and that coding variants have the highest enrichment levels (i.e. have the largest effects on average). The high contribution of regulatory variants is consistent with the findings of Xiang et al. [35], who recently estimated that gene expression and RNA splicing explain large proportions of the heritability for complex traits. Therefore, we will place more emphasis on coding and regulatory variants in the strategies evaluated. A particular focus will be on putative regulatory elements detected in muscle, as the breed is primarily selected for muscular development.

## Methods

### Data

Our study used a cohort of 18,324 BBC genotyped cows that we imputed at the sequence level. The genotyping data and methodology are very similar to those described in Gualdrón Duarte et al. [33], where further details can be found. Briefly, cows were genotyped on 10 distinct genotyping arrays, including five versions of the Illumina Bovine Low Marker Density (LMD) genotyping arrays (ranging from 9077 to 16,381 SNPs) and five versions of the EuroGenomics Medium Marker Density (MMD) arrays (ranging from 48,699 to 68,454 SNPs). The number of individuals per array are reported in Additional file 1: Table S1 (only individuals with a call rate > 0.90 were selected for this study).

The phenotypes included four linear classification scores, that assessed the muscular development of the shoulder, top and buttocks (rear and side view) of the animals on a scale of 0–50. To derive the overall score for muscular development, the individual scores were combined with different weights (1 for top and shoulder muscling, 2 for buttock muscling). These phenotypes, available for 16,508 of the cows, were corrected for fixed effects from the official genetic evaluation as described in Gualdrón Duarte et al. [33]. Two reference panels of bulls were available for genotype imputation, including a group of 717 AI bulls genotyped with the Illumina BovineHD genotyping array and whole-genome sequence data from 230 bulls. Details of the bioinformatic analysis of the sequence data, including read mapping and variant calling and filtering, can be found in Gualdrón Duarte et al. [33]. The final Variant Calling file (VCF) from the 230 sequenced bulls included 15,332,952 variants (12,830,339 SNPs and 2,502,613 indels). From these, we selected only bi-allelic autosomal variants.

### Genotype imputation

A multi-step genotype imputation procedure was applied. First, SNP filtering was performed separately for each LMD and MMD array. SNPs with low call rate (< 0.95), with minor allele frequency (MAF) < 0.01 or with significant deviations from Hardy–Weinberg proportions (p > 0.001) were filtered out. We first performed imputation from the LMD arrays to the MMD level, one array at a time. The MMD panel consisted of all individuals genotyped on one of the five MMD arrays. After filtering SNPs based on the rules described above, 36,849 autosomal markers with MAF > 0.01 and a maximum of 5 Mendelian inconsistencies in duos or trios, common to these five arrays and also present on commercial Illumina MMD arrays were retained to define the reference MMD panel. The different LMD arrays had 7246, 7505, 7711, 7632 and 7775 SNPs in common with the reference MMD panel, respectively. The target and reference panels were then phased using ShapeIT4.2 [8] and imputation in the target panel was achieved using Minimac4 [5]. After imputation, we excluded markers with a MAF < 0.02 or an imputation accuracy below 0.90 (for each array separately), and MMD genotypes from all individuals were merged. After selecting markers shared with the High Marker Density (HMD) reference panel, 31,112 were available for the second imputation step. We used the HMD reference panel previously prepared by Gualdrón Duarte et al. [33], which contained 890 individuals (717 genotyped and 173 sequenced bulls) and 611,322 markers. The same imputation and filtering procedure was applied as in the first imputation step. Finally, the cows were imputed to the sequence levels using 578,934 markers and the reference panel of 230 sequenced bulls from the study of Gualdrón Duarte et al. [33]. After this last imputation step, we selected variants imputed with imputation accuracy > 0.90, MAF > 0.01 and segregating according to HWE rules (p > 0.001), leaving 11,280,414 autosomal bi-allelic SNPs and indels for subsequent analyses.

### Genomic prediction models

#### General genomic prediction models

We first describe models that don't use functional annotation, including GBLUP and three Bayesian models. In this case, the annotation information can be used, for example, to pre-select the variants to be included in the genomic prediction models.

In the GBLUP model, phenotypes are modelled as:

$$\mathbf{y}=\mathbf{1}\upmu +\mathbf{g}+\mathbf{e},$$

where **y** is the vector of individual phenotypes, $\mathbf{1}$ is a vector of 1’s, $\upmu$ is the mean effect, **g** is the vector of individual polygenic terms, and **e** is the vector of individual independent random error terms, normally distributed, $\mathbf{e} \sim N(\mathbf{0},{\mathbf{I}\upsigma }_{\text{e}}^{2})$ where **I** is the identity matrix and ${\upsigma }_{\text{e}}^{2}$ is the residual variance. The polygenic effects are normally distributed, $\mathbf{g} \sim N(\mathbf{0},{\mathbf{G}\upsigma }_{\text{g}}^{2})$ where **G** is the GRM and ${\upsigma }_{\text{g}}^{2}$ is the variance of polygenic effects. The GRM can be computed using the matrix **Z** of centered genotypes, corresponding to the first rules proposed by VanRaden [36] and assuming that the distribution of SNP effect does not depend on allele frequencies:

$$\mathbf{G}=\frac{\mathbf{Z}\mathbf{Z}^{\prime}}{\sum_{j=1}^{\text{N}}2f_j(1-f_j)},$$

where *f*_*j*_ is the allele frequency at marker *j* and N is the number of markers. Alternatively, the GRM can be obtained using the matrix **X** of centered and scaled (or “standardized”) genotypes as described in Yang et al. [37]:

$$\mathbf{G}=\frac{\mathbf{X}\mathbf{X}^{\prime}}{\text{N}}.$$

In this case, rare alleles have larger effects and all variants contribute equally to the genetic variance. We will use GBLUP-C and GBLUP-S to refer to GBLUP with centered and standardized genotypes, respectively. The GRM and GBLUP prediction calculations were performed using LDAK [38]. For the GBLUP model, the variance components were estimated using a restricted maximum likelihood (REML).

For the Bayesian models, phenotypes are described as:

$$\mathbf{y}=\mathbf{1}\upmu +\mathbf{Z}{\varvec{\upbeta}}+\mathbf{e},$$

where **β** is the vector of SNP effects. The models can be applied with centered or standardized (replacing **Z** by **X**) genotypes. A key difference between the Bayesian models is the distribution of SNPs effects ${\upbeta }_{\text{j}}$. In BayesCπ [39], a fraction π of SNPs have a null effect:

$${\upbeta }_{\text{j}}\sim\uppi {\updelta }_{0}+\left(1-\uppi \right)N\left(0,{\upsigma }_{\upbeta }^{2}\right),$$

where δ_0_ is a discrete probability mass at 0. The proportion of SNPs with zero effect (π) and the common variance of SNP effects ${\upsigma }_{\upbeta }^{2}$ are estimated from the data. BayesCπ was run using the GCTB software [40] with default settings.

In the Bayesian Sparse Linear Mixed Model (BSLMM) [41], SNP effects are also distributed as a mixture of two distributions, with all SNPs having at least a small effect and a few SNPs having an additional effect:

$${\upbeta }_{\text{j}}\sim\uppi N\left(0,{{\upsigma }_{\text{a}}^{2}+\upsigma }_{\text{b}}^{2}\right)+\left(1-\uppi \right)N\left(0,{\upsigma }_{\text{b}}^{2}\right),$$

where ${\upsigma }_{\text{b}}^{2}$ is the variance of small effects, ${\upsigma }_{\text{a}}^{2}$ is the variance associated with additional effects. The parameter π is now the proportion of SNPs with additional effects. As in BayesCπ, the parameters are estimated. The model is implemented by modelling a polygenic term and using the associated GRM. BSLMM was run using the GEMMA software [41] with default settings.

Finally, in BayesR [17], the SNP effects are sampled from a mixture of four distributions:

$${\upbeta }_{\text{j}}\sim {\uppi }_{1}{\updelta }_{0}+{\uppi }_{2}N\left(0,{10}^{-4}{\upsigma }_{\text{g}}^{2}\right)+{\uppi }_{3}N\left(0,{10}^{-3}{\upsigma }_{\text{g}}^{2}\right)+{\uppi }_{4}N\left(0,{10}^{-2}{\upsigma }_{\text{g}}^{2}\right),$$

where π_1_, π_2_, π_3_ and π_4_ are the proportions of SNPs in the four categories. Where π_1_ is the proportion of SNPs with null effects and π_4_ is the proportion of SNPs with the largest effects, corresponding to one percent of the polygenic variance. The variances associated with each category are predetermined as fixed proportions of the polygenic variance which is estimated from the data as the mixture proportions. BayesR was run using the GMRM software [20] without annotation (see below for more information).

#### Genomic predictions models exploiting prior biological information

Two methods were applied to perform whole genome predictions using directly information from functional annotations. For this purpose, each SNP is assigned to one of the annotation groups, referred to as genomic features (GFs) to align with the terminology used in the literature [14, 15], described in the next section. First, we used a GBLUP, in which a distinct polygenic term is defined for each GF. The principle is similar to the MultiBLUP model described by Speed and Balding [16] and the GFBLUP which fits most often a single GF at a time. We therefore call this model a Multiple Genomic Feature BLUP (MGFBLUP), and implement it as follows:

$$\mathbf{y}=\mathbf{1}\upmu +\sum\nolimits_{\text{s}=1}^{\text{S}}{\mathbf{g}}_{\text{s}}+\mathbf{e},$$

where **g**_s_ is the vector of individual polygenic terms associated to GF s, S is the total number of fitted GF. Each polygenic component is normally distributed, ${\mathbf{g}}_{\text{s}} \sim N(0,{{\mathbf{G}}_{\text{s}}\upsigma }_{\text{s}}^{2})$ where **G**_s_ is the GRM computed using the variants present in GF s and ${\upsigma }_{\text{s}}^{2}$ is the variance of polygenic effects from the GF. As for the GBLUP, centered or standardized GRM can be used (MGFBLUP-C versus MGFBLUP-S). The genetic parameters, including the variances associated with each GF and the residual variance, were estimated using a REML approach as implemented in LDAK [38].

The second approach is a Bayesian grouped mixture of regressions model (GMRM), also called BayesRR-RC [20] and derived from BayesR [17] and BayesRC [18]. In this model, phenotypes are described as:

$$\mathbf{y}=\mathbf{1}\upmu +\sum_{\text{s}=1}^{\text{S}}{\mathbf{X}}_{\text{s}}{{\varvec{\upbeta}}}_{\text{s}}+\mathbf{e},$$

where **X**_s_ is the matrix of centered and scaled genotypes for markers in GF s and **β**_s_ is the vector of marker effects for GF s, that are modelled as a mixture of null effects (spike probability at zero) and Gaussian distributions:

$${\upbeta }_{{\text{s}}_{\text{j}}}\sim {\uppi }_{{0}_{\text{s}}}{\updelta }_{0}+{\uppi }_{{1}_{\text{s}}}N\left(0,{\upsigma }_{{1}_{\text{s}}}^{2}\right)+{\uppi }_{{2}_{\text{s}}}N\left(0,{\upsigma }_{{2}_{\text{s}}}^{2}\right)+{\dots +\uppi }_{{\text{L}}_{\text{s}}}N\left(0,{\upsigma }_{{\text{L}}_{\text{s}}}^{2}\right),$$

where j is the marker index, L is the number of Gaussian distributions in the mixture, $\left\{{\uppi }_{{0}_{\text{s}}},{\uppi }_{{1}_{\text{s}}}, {\uppi }_{{2}_{\text{s}}}, \dots ,{\uppi }_{{\text{L}}_{\text{s}}}\right\}$ are the mixture proportions for GF s, $\left\{{\upsigma }_{{1}_{\text{s}}}^{2},{\upsigma }_{{2}_{\text{s}}}^{2}, \dots ,{\upsigma }_{{\text{L}}_{\text{s}}}^{2}\right\}$ are the mixture variances for GF s, proportional to ${\upsigma }_{\text{s}}^{2}$, the variance explained by the GF. Here, L is equal to 3, with variances ${\upsigma }_{{\text{l}}_{\text{s}}}^{2}$ equal to 0.0001, 0.001 and 0.01 ${\upsigma }_{\text{s}}^{2}$, respectively. The hyper-parameters vary for variants from different GFs, and the variances ${\upsigma }_{\text{s}}^{2}$ are estimated from the data. This model was run using the GMRM software [20] with a Gibbs sampling scheme for 5000 iterations with a burn-in period of 2000 iterations. This setting corresponds to the values used by Patxot et al. [20] and Orliac et al. [23]. When BayesRR-RC is used without annotation, we will refer to it as a BayesR model.

With these two approaches exploiting functional annotation, it is possible to define two parameters related to the contribution of a category to heritability and the relative size of effects in a category. First, the proportion of genetic variance explained by a category, also called percentage of heritability or %SNP heritability [42], is estimated as ${\upsigma }_{\text{s}}^{2}$ divided by ${\upsigma }_{\text{g}}^{2}$. Second, the enrichment level in category i, has been defined by Gusev et al. [42] as the percentage of heritability in category i divided by the proportion of variants in the same category.

### Annotation

We considered protein-coding variants to be those that alter the protein (e.g. change in amino acid sequence, truncations, alternative splice sites). To identify such variants, we ran Variant Effect Prediction (VEP) v95.0 [43] on our VCF file. The most common coding consequences were missense, splice site (donor and acceptor), frameshift and stop-gain variants. VEP also provides the predicted effect from the variants, which is MODERATE or HIGH for coding variants and MODIFIER or LOW for other variants. Therefore, this category of protein-coding variants includes all the variants with the highest predicted impacts.

We used three sources of information to identify putative regulatory variants. EQTLs provide the most direct evidence, as these variants present significant association with expression levels. Therefore, we extracted all cis-eQTLs from the cattle Genotype-Tissue Expression atlas (cGTEx) data base [44]. For each eQTL we selected the lead SNP. This resulted in the selection of 22,817 eQTLs, including 4889 eQTLs identified in muscle. In addition, variants located in open chromatin regions represent potential regulatory variants. Therefore, we used the catalogue of regulatory elements detected by the assay for transposase accessible chromatin using sequencing (ATAC-seq) generated by Yuan et al. [45]. This organism-wide catalogue contains 976,813 cis-acting regulatory elements in 68 bovine tissues types. Variants located in these peaks represented 10% of the genome space. Finally, regulatory elements identified by Kern et al. [46] in eight tissues, including muscle, were also considered as possible regulatory variants. These regulatory elements were identified thanks to epigenetic data for four histone modifications and one DNA binding protein (CTCF), and by applying ChromHMM [47] to predict genome-wide chromatin states in each tissue. Among the identified states, we selected active regulatory element states, including “CTCF / Active TSS”, “Active TSS”, “CTCF / promoters”, “Active promoters”, “CTCF / enhancers” and “Active enhancers”, where TSS stands for transcription start sites. All of these active marks are associated with the co-occurrence of at least two histone modifications and/or CTCF binding, and broad marks (e.g. associated only with the histone modification H3K27me3) were excluded.

We relied on the General Transfer Format (GTF) file of the bovine genome assembly available from Ensembl (v105) to classify the remaining variants. First, TSS and transcription termination sites (TTS) were obtained using Homer [48] and all transcripts from the genes. Upstream and downstream regions were then defined as 1 kb upstream and downstream of the TSS and TTS, respectively. Variants were then classified into three additional groups including “Exon-associated elements” (encompassing exons and neighboring regions such as untranslated regions (UTRs) and regions upstream or downstream of genes), intronic regions, and intergenic regions corresponding to the remaining unannotated regions. Note that the Exon-associated elements contain only non-coding variants (e.g. synonymous variants) and putative regulatory regions that were not detected by the functional assays (i.e. not in the eQTL or regulatory element lists). There is in fact a hierarchy between the defined groups; if a variant can be associated with more than one group, we have chosen the group with the highest expected effect. The ranking of the groups, from most to least impactful, includes coding variants, eQTLs, regulatory elements (identified by ATAC-seq or with epigenetic data), exon-associated elements, intronic and intergenic variants.

### Experimental design

To assess the prediction accuracy of different models, we performed a cross-validation analysis and divided our data set into a reference and a target population corresponding to 13,461 and 3047 cows born before and after 1st January 2019, respectively. We then applied the different models with different marker panels and using different annotation groups. In most cases, we limited the number of annotation groups to 8 because more groups could lead to convergence problems with REML (or to null variances). Reliability was obtained as the squared correlation between genomic estimated breeding values (GEBV) and trait deviations divided by the heritability of the trait.

As done by Meuwissen et al. [49], we used a bootstrapping strategy to evaluate the significance of the difference in reliability between different methods or when using different marker panels. We created a table with the GEBVs of the > 3000 target individuals (rows) for all tested methods (columns). We then sampled the validation individuals with replacement 10,000 times and estimated the correlation between GEBVs and trait deviations, and estimated the reliability or reliability difference for each sample. The 2.5th and 97.5th quantiles were used to define the confidence intervals. Differences were considered significant if one method was higher in 97.5% or more of the samples.

#### Genomic prediction using whole-genome sequence data

We started by using all 11,280,414 variants available at the sequence level without annotation and ran centered and standardized GBLUP and BayesR. BayesCπ and BSLMM were not run on the full sequence for computational reasons. Next, we defined a first functional annotation model with eight groups (FAN1): coding variants, eQTLs, variants in regulatory elements identified by both ATAC-seq and with epigenetic data, variants in regulatory elements detected with epigenetic data only, variants in regulatory elements detected by ATAC-seq only, exon-associated elements, intronic regions and intergenic regions. With the second annotation model, we investigated whether separating regulatory variants identified in muscle from those identified only in other tissues improved prediction. In this case, the putative regulatory variants group contained variants in regulatory elements identified by ATAC-seq or with epigenetic data. This resulted in the following eight annotation groups (FAN2): coding variants, muscle eQTLs, other eQTLs, variants in muscle regulatory elements, variants in other regulatory elements, exon-associated elements, intronic and intergenic regions. In addition, we tested whether a stratification model based on LD and MAF (LDMS) improved prediction accuracy, as Orliac et al. [23] have shown that these groups are important to include. We defined three MAF categories (0.01 < 0.05; 0.05–0.10; 0.10–0.50) and four LD-based categories (defined based on the LD score quartiles). These LD scores were calculated using GCTA [37]. We also combined the LDMS and FAN1 models, resulting in 8 × 12 groups. Note that this last model was only run with GMRM [20], as we previously observed that the REML approach often had convergence problems when fitting a model with 12 or more groups [34]. These different models are described in Table 1, including the (functional) groups fitted in the models, and the number of variants per group.

**Table 1:** Description of the different annotation models and their respective categories

Model	Annotation group	Number of variants per group	Proportion in the genome (%)
FAN1: eight functional annotation groups allowing distinct effect sizes for coding and regulatory variants	Coding variants	41,866	0.37
	eQTLs	31,521	0.28
	Regulatory elements detected by ATAC-seq	855,103	7.58
	Regulatory elements detected with epigenetic data	431,616	3.83
	Regulatory elements detected with both techniques	333,877	2.96
	Exon-associated elements	732,544	6.49
	Intronic	2,994,362	26.54
	Intergenic	5,859,525	51.94
FAN2: eight functional annotation groups similar to FAN1 but with specific categories for regulatory elements detected in muscle	Coding variants	41,866	0.37
	eQTLs detected in muscle	4761	0.04
	eQTLs detected in other tissues	26,760	0.24
	Regulatory elements detected in muscle	80,378	0.71
	Regulatory elements detected in other tissues	1,540,218	13.65
	Exon-associated elements	732,544	6.49
	Intronic	2,994,362	26.54
	Intergenic	5,859,525	51.94
LDMS: 12 groups based on the combination of four LD groups based on LD score and three MS groups based on MAF values	LD: Four equal groups based on LD score quartiles	2,820,104	25.00
	MS: Minor allele frequency between 0.01 and 0.05	2,193,621	19.45
	MS: Minor allele frequency between 0.05 and 0.10	1,663,801	14.75
	MS: Minor allele frequency between 0.10 and 0.50	7,422,992	65.80
LDMS x FAN1: interaction between FAN1 and LDMS model	96 groups based on the combination of the 12 LDMS groups and the 8 FAN1 groups	From 29 to 1,446,999	From 0.00 to 12.83

Finally, the BayesRR-RC model was run twice with the FAN1 model to assess the variability in heritability partitioning across functional classes and prediction accuracy. In order to identify possible confounding between classes, we computed the correlations between the variances estimated in different iterations.

#### Use of biological information to pre-select markers

We then used the functional annotation groups to pre-select variants from the WGS data, as has been done in several studies [18, 28, 50, 51]. This is an indirect approach to include biological information with models that can’t incorporate it directly (GBLUP, BayesCπ, BayesR and BSLMM), and amounts to assume that variants in unselected categories have a null effect. More importantly, it allows the data set and computational costs to be reduced, thus allowing the use of other models such as BayesCπ and BSLMM. Here, we selected a large subset of markers. This was done to include a high proportion of coding and regulatory variants and still capture the majority of sequence-level variants through LD.

Our first selection (Panel FUN1) included markers from the MMD panel currently used in the genomic evaluation and all coding and putative regulatory variants, including eQTLs and variants in regulatory elements detected by ATAC-seq or with epigenetic data, resulting in a selection of 1,715,587 variants. We also defined a second panel (Panel FUN2) with fewer markers. It was generated using the same rules as above, except that putative regulatory elements were identified only based on the open chromatin regions defined by Yuan et al. [45]. This amounts to using only one catalogue of putative regulatory elements and resulted in a panel with 1,284,915 markers. Similarly, we defined Panel-FUN3 using instead the catalogue of regulatory elements from Kern et al. [46] and obtained 863,615 markers. For comparison, we generated other panels obtained by performing LD pruning (based on the r^2^ measure) with thresholds of 0.99, 0.98, 0.95, 0.90 and 0.80, resulting in selection of 1,899,123, 1,708,694, 1,436,932, 1,203,927 and 923,968 variants, respectively (Panels LD99 to LD80). In addition, we selected all markers present on commercial bovine genotyping arrays extracted from the SNPchiMp data base [52], resulting in 868,195 polymorphic SNPs (Panel ARRAY). Table 2 summarizes all the defined panels, their size and how the markers were selected.

**Table 2:** Description of the different selected marker panels

Panel	Selection criteria	Number of variants
WGS	All whole-genome sequence variants	11,280,414
FUN1	Coding variants, eQTLs, variants in regulatory elements detected by ATAC-seq or epigenetic data and markers on MMD array	1,721,775
FUN2	Coding variants, eQTLs, variants in regulatory elements detected by ATAC-seq only and markers on MMD array	1,292,091
FUN3	Coding variants, eQTLs, variants in regulatory elements detected by epigenetic data only and markers on MMD array	870,858
LD99	Selection based on LD pruning with a threshold of r^2^ > 0.99	1,899,123
LD98	Selection based on LD pruning with a threshold of r^2^ > 0.98	1,708,694
LD95	Selection based on LD pruning with a threshold of r^2^ > 0.95	1,436,932
LD90	Selection based on LD pruning with a threshold of r^2^ > 0.90	1,203,927
LD80	Selection based on LD pruning with a threshold of r^2^ > 0.80	923,968
ARRAY	Variants from different commercial bovine genotyping arrays	868,195

For all panels, we first ran models without annotation, including centered and standardized GBLUP, BayesCπ, BSLMM and BayesR. For panels FUN1, FUN2 and FUN3, we ran BayesRR-RC and MGFBLUP with similar functional groups as for the sequence data, but adapted as some categories were removed from the data. FAN1 now contained four groups (MMD markers, coding variants, eQTLs, variants in regulatory elements) while FAN2 still contained six groups (MMD markers, coding variants, muscle eQTLs, other eQTLs, variants in muscle regulatory elements, variants in other regulatory elements).

## Results

### Genomic prediction models using all sequence-level variants

**Fig. 1:**
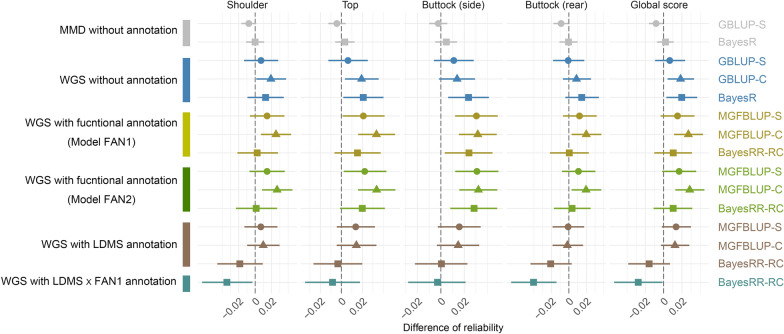
Gain of reliability obtained when using whole-genome sequence data, with or without functional annotation. The gain in reliability compared to a GBLUP-C model with a medium marker density (MMD array). GBLUP and BayesR correspond to models without functional annotation, while the Multiple Genomic Feature BLUP (MGFBLUP) and BayesRR-RC refer to extensions of these models that make use of functional annotation. For the GBLUP and MGFBLUP models, the extensions ‘-C’ and ‘-S’ indicate whether the used GRMs were constructed with centered and standardized genotypes, respectively. Genomic predictions were performed using different panels (MMD and whole-genome sequence—WGS) and models including models without annotation, two models incorporating functional annotation (FAN1 and FAN2), a model based on LD and MAF stratification (LDMS), and a combination of LDMS and FAN1 models. Further details of these models are provided in Table 1. Error bars indicate the 95% confidence interval of the bootstrapped differences

Reliabilities obtained with GBLUP-C using the MMD panels were 0.792, 0.674, 0.686, 0.750 and 0.705 for shoulder, top, buttock (side view), buttock (rear view) and overall muscling, respectively. As the GBLUP-C is the approach currently used in genomic evaluation, we used these as the baseline or reference values and presented results from models using the full sequence data as the difference from these baseline values (Fig. 1). We first evaluated models using the 11 million imputed variants (Fig. 1; see Additional file 2: Table S8). This was only possible with GBLUP (without annotation) and MGFBLUP (fitting one polygenic term per annotation group) approaches using either centered or standardized genotypes, and with the GMRM program fitting the BayesR (without annotation) and BayesRR-RC (with annotation) models. Compared to MMD arrays, the use of WGS data consistently resulted in higher accuracy for the three annotation-free models and for all traits (see Additional file 2: Table S8). With the GBLUP, the use of centered genotypes (GBLUP-C) gave better results (+ 1.6%, i.e. 1.6 percentage points, reliability on average) than standardized genotypes (GBLUP-S) (+ 0.6% on average). Prediction accuracies achieved with BayesR (+ 1.8% on average), implemented using standardized genotypes, were systematically higher than those obtained with GBLUP-S, suggesting advantages of the Bayesian approach. However, the superiority was less pronounced when compared with GBLUP-C. Although these trends were consistent across traits, only a few of these differences were significant (see Additional file 3: Figure S1).

Compared to GBLUP, the reliabilities obtained using functional annotation with the MGFBLUP approach were higher (Fig. 1), although the differences were rarely significant (see Additional file 3: Figure S1). For example, with centered genotypes, the MGFBLUP reliabilities were on average + 1.2% higher for the two functional annotation models tested (FAN1 and FAN2). The opposite trend was observed for the Bayesian models, the reliabilities of BayesRR-RC with the FAN1 and FAN2 models being on average − 0.8% and − 0.6% lower than those obtained with BayesR (without annotation). When groups were defined based on LD and MAF (LDMS models), lower reliabilities were obtained with MGFBLUP-C (− 1.6% on average) and BayesRR-RC (− 3.0% on average) compared to the corresponding models without annotation. Reliabilities obtained with BayesRR-RC were particularly low when fitting 96 groups with the LDMS x FAN1 model (− 4.1% on average). Overall, of all the methods tested, the MGFBLUP-C model with functional annotation achieved the highest accuracies for each of the traits.

**Fig. 2:**
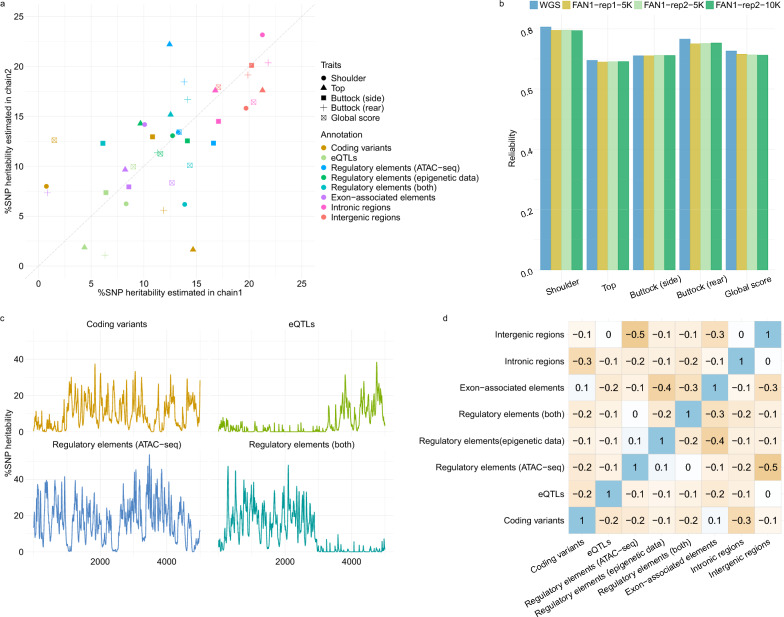
Comparison of results of BayesRR-RC with two independent chains. **a** Percentage heritability (%SNP heritability) of different functional categories estimated in two independent runs. **b** Reliability of GS in the two independent chains for the FAN1 model compared to accuracy with WGS (without annotation); results are also reported with 10,000 iterations for the second chain (instead of 5000). **c** Evolution of estimated parameters over iterations for four functional categories. **d** Correlations between estimated parameters for different functional categories in different iterations (when estimated for buttock – side view)

The proportion of variance allocated to different functional categories and the enrichment levels of variants with different annotations allow a better understanding of how the models use the functional information. For example, estimated parameters from the first model (FAN1) using functional annotation (Table 3 for average values and see Additional file 1: Tables S2–S6 for values per trait) showed that coding variants and eQTLs had larger effects per SNP on average (average enrichment levels above 15-fold and 20-fold, respectively), followed by variants in putative regulatory elements with average enrichment levels ranging from 1.7 to 3.9-fold depending on the method (Table 3). With MGFBLUP models, eQTLs had even larger effects than coding variants (e.g. 16.1-fold versus 70.5-fold when using centered genotypes). Nevertheless, these relatively small groups (each containing less than 0.4% of the variants) together accounted for only 10–25% of the genetic variance, whereas intergenic and intronic variants still accounted for a large proportion of the genetic variance (about 40%), as together they represent more than 75% of the variants in our data set. With MGFBLUP and the second annotation model (FAN2), variants associated with eQTLs or regulatory elements detected in muscle had higher enrichment levels than variants in the same elements detected in other tissues (e.g. muscle eQTL enrichment levels were on average higher than 100-fold), whereas the opposite was observed for eQTLs with BayesRR-RC (Table 3). Some unexpected results were observed, such as reduced enrichment levels for coding variants with the FAN2 model and BayesRR-RC, or a null variance associated with the category of exon-associated elements when estimated with MGFBLUP (Table 3). Such results indicate that parameters can be difficult to estimate (see also Yuan et al. [34]) and that enrichment levels used in predictions may not always reflect true biological enrichment levels. The variation in estimated parameters across traits (see Additional file 1: Tables S2–S6; Fig. 2a) confirmed this technical difficulty. Interestingly, this variation had little effect on the relative performance of the different models. To further understand aspects of convergence with BayesRR-RC, we ran an additional independent chain for the FAN1 model (including more iterations), generated some diagnostic plots, and assessed the level of confounding between categories from their correlation across iterations (Fig. 2a–d). Estimated genetic variances for different functional categories showed differences across independent runs (Fig. 2a), particularly for coding variants, while diagnostic plots suggested that convergence may not have been achieved for all parameters (Fig. 2c), possibly due to confounding between some parameters (e.g. between intergenic variants and variants in regulatory elements detected by ATAC-seq categories; Fig. 2d). Despite these differences in estimated enrichment levels, similar prediction accuracies were obtained with the two independent chains and when more iterations were run (Fig. 2b).

**Table 3:** Average estimated %SNP heritability (proportion of genetic variance explained by a category) and enrichment levels (relative variant effect size per category) for different functional categories with the two functional annotation models, FAN1 and FAN2

	Annotation group (compartment where variants are located)	%SNP heritability			Enrichment		
		MGFBLUP Centered	MGFBLUP Standardized	BayesRR-RC	MGFBLUP Centered	MGFBLUP Standardized	BayesRR-RC
FAN1	Coding variants	5.98	7.65	7.99	16.11	20.61	21.54
	eQTLs	14.91	17.33	4.36	70.50	81.93	20.59
	Regulatory elements detected with both techniques	7.87	8.21	11.59	2.65	2.77	3.91
	Regulatory elements detected by ATAC-seq	20.48	12.96	13.13	2.70	1.71	1.73
	Regulatory elements detected with epigenetic data	8.74	6.89	11.74	2.28	1.80	3.06
	Exon-associated elements	0.22	0.07	7.09	0.03	0.01	1.09
	Intronic regions	26.87	31.11	21.60	1.01	1.17	0.81
	Intergenic regions	14.94	15.78	22.49	0.29	0.30	0.43
FAN2	Coding variants	5.88	8.03	3.45	15.84	21.64	9.28
	eQTLs detected in muscle	4.57	4.71	0.24	108.22	111.56	5.71
	eQTLs detected in other tissues	11.28	12.72	5.82	57.89	65.27	29.86
	Regulatory elements detected in muscle	9.35	9.52	13.69	13.11	13.35	19.19
	Regulatory elements detected in other tissues	24.46	18.14	19.99	1.79	1.33	1.46
	Exon-associated elements	0.49	0.10	12.26	0.08	0.02	1.89
	Intronic regions	27.15	30.24	22.03	1.02	1.14	0.83
	Intergenic regions	16.83	16.55	22.53	0.32	0.32	0.43

MGFBLUP models were applied with GRMs constructed with either centered or standardized genotypes

### Genomic prediction models using subsets of the sequence data

**Fig. 3:**
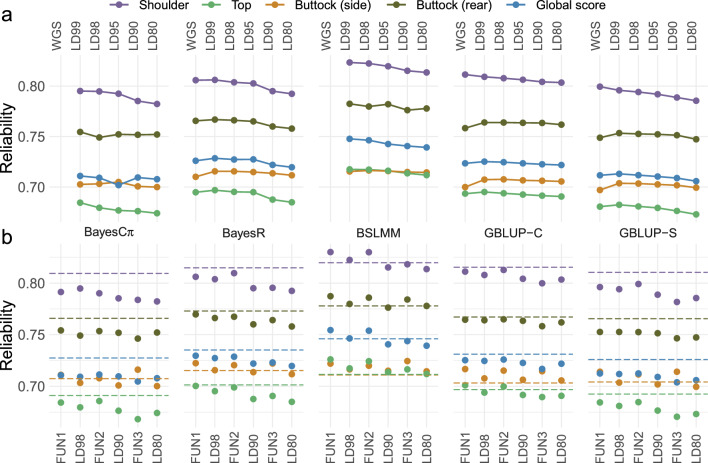
Comparison of reliability of different methods when using different marker panels.** a** Comparison of the reliability of five tested methods using panels selected on the basis of LD pruning. The panels are whole-genome sequence (WGS—no pruning) and LD99, LD98, LD95, LD90 and LD80 obtained when pruning was applied with thresholds of r^2^ > 0.99, 0.98, 0.95, 0.90 and 0.80, respectively. **b** Reliability obtained using panels selected on the basis of functional annotation. The panels included coding variants, eQTL, variants in regulatory elements and markers from the medium density genotyping array. The FUN1 panel included variants in regulatory elements detected by ATAC-seq or epigenetic data, while the FUN2 and FUN3 panels include only those detected by either ATAC-seq or epigenetic data, respectively. The results were compared to those obtained with panels of equal size selected by LD pruning. The horizontal dashed line corresponds to the reliability obtained with the ARRAY panel, obtained by selecting markers present on commercial bovine genotyping arrays, with approximately the same number of variants as the FUN3 panel. Further details on the different panels and their size are given in Table 2

We compared different strategies for selecting large subsets of the sequence data, large enough to still capture the full sequence level while allowing the use of additional, more computationally demanding software, including GCTB for BayesCπ [39] and GEMMA for BSLMM [41]. For all methods, the highest accuracy was achieved in the vast majority of cases with an LD pruning level of r^2^ > 0.99 (1.9 M variants) (Fig. 3a; see Additional file 2: Table S9). Reliability was even higher than with full sequence data (for BayesR and GBLUP approaches). Reliabilities decreased only slightly when stronger LD pruning was applied and the number of variants was further reduced (< 1% on average). For traits such as buttock muscling (side view), the reliabilities remained almost the same even when using an LD pruning level of r^2^ > 0.80 (0.9 M variants), whereas the greatest reduction in reliability was observed for shoulder muscling. However, the differences between the largest and smallest marker panels were not always significant, depending on the method (see Additional file 3: Figure S2).

At all LD pruning levels, BSLMM was systematically the best approach (with a single exception, BayesR achieving slightly higher reliabilities for buttock side when using a LD pruning level of 0.99), while BayesCπ and GBLUP-S were often the least accurate (Fig. 3a). The ranking between the BayesR and GBLUP approaches was consistent with that observed with the full sequence data, BayesR achieving on average higher reliability than both GBLUP approaches (for LD pruning levels of 0.95 or higher) and GBLUP-C being superior to GBLUP-S (Fig. 3a). At the r^2^ > 99 pruning level, the accuracies obtained with BSLMM were significantly higher than those achieved with BayesR, GBLUP-C, GBLUP-S, and BayesCπ for several traits (with the exception of buttock side with BayesR; Fig. 3a; see Additional file 3: Figure S3).

With BSLMM, the average number of variants with an additional effect fitted in the model ranged from 22 to 138 (mean = 71.0) per trait (LD99), while with BayesR and BayesCπ, the average number of variants with a non-zero effect ranged from 10,990 to 11,507 (mean = 11,193) and 121,759 to 124,891 (mean = 135,818), respectively (LD99). With BayesR, the number of variants with a medium or large effect (0.001 and 0.01 × ${\upsigma }_{\text{g}}^{2}$) were low, 3.8 and 1.5 on average. Note that the different categories of variants are not directly comparable between these two methods. With stronger LD pruning, the number of additional effect variants increased slightly with BSLMM (85.8 and 105.4 on average for LD98 and LD80, respectively), while the proportion of non-zero effects (corresponding to π) remained relatively stable with BayesCπ (π remained close to 0.05). This was also the case for the number of medium and large effect variants with BayesRR-RC (e.g., 3.9 and 0.8 for LD80).

**Fig. 4:**
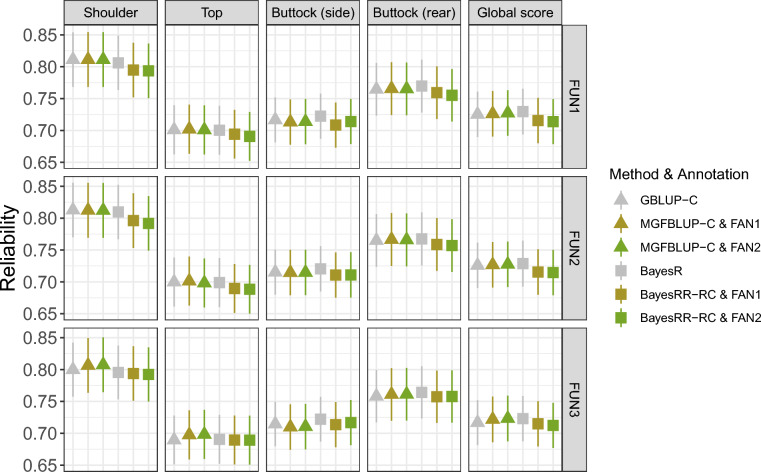
Reliability of models incorporating functional annotation applied to subsets of markers. Results were obtained using the three functional panels (FUN1-3) and applying GBLUP-C or BayesR without functional annotation or with the Multiple Genomic Feature BLUP (MGFBLUP) and BayesRR-RC models with the two functional models (FAN1 and FAN2 models). The FUN1 panel (top) includes variants in regulatory elements detected by ATAC-seq or epigenetic data, while the FUN2 (middle) and FUN3 (bottom) panels include only those detected by either ATAC-seq or epigenetic data, respectively. The FAN1 model has four groups, including coding variants, eQTLs, variants in regulatory elements and markers from the medium density genotyping array. In the FAN2 model, two additional categories are obtained by dividing eQTLs and variants in regulatory elements into those detected in muscle and those detected in other tissues. Details of the models and marker panels are given in Tables 1 and 2

We then compared the reliabilities obtained with marker panels selected based on functional annotation (called FUN1-3 panels) with panels of equivalent size selected based on LD pruning (LD panels) or panels containing markers present on commercial bovine genotyping arrays (870K SNPs) (Fig. 3b; see Additional file 2: Table S9). For most methods, marker selection based on functional annotation resulted in slightly higher or equivalent accuracy than LD-based marker selection (Fig. 3b), although the differences were almost never significant when compared at equal density (see Additional file 3: Figure S4). In particular, the FUN1 and FUN2 panels were often more efficient than the corresponding LD panels. In BSLMM, the advantage of the FUN panels was observed for all traits and marker sizes. Higher reliabilities were systematically obtained with the ARRAY panel when using GBLUP-S or BayesCπ (except for buttock—side view), while for GBLUP-C and BayesR the accuracy was very close to that obtained with the two largest FUN panels. Importantly, the ARRAY panel was in most cases significantly superior to other panels of equivalent size for these four methods. With BSLMM, the use of the FUN1 and FUN2 panels resulted in higher reliabilities than the use of the ARRAY panel, while for the FUN3 panel the reliabilities obtained were either higher or equal to those obtained with the ARRAY panel (Fig. 3b). Note that the use of FAN1 and FAN2 models incorporating functional annotation (BayesRR-RC and MGFBLUP) with the FUN1 and FUN2 panels did not improve the reliability of genomic prediction, while at best a slight improvement was observed when using the FUN3 panel (Fig. 4; see Additional file 2: Table S10).

Overall, the highest reliabilities were obtained using BSLMM with variants selected based on their functional annotation, closely followed by BSLMM with LD panels. Compared with GBLUP-C using MMD markers, the reliabilities obtained with BSLMM for the FUN1 panel were significantly 0.052, 0.038, 0.036, 0.038 and 0.049 higher for top, shoulder, buttock (side and rear view) and overall muscling, respectively (see Additional file 1: Table S7). Similar benefits were also obtained with the LD99 panel (see Additional file 1: Table S7). With the FUN1 and FUN2 panels, the number of larger effect variants ranged from 30 to 91 (mean = 63.9) and from 33 to 200 (mean = 86.2), respectively. The %SNP heritability associated with the additional effect variants was 14.3% and 16.2% on average with the FUN1 and FUN2 panels, respectively. We investigated which regions contained variants with high posterior inclusion probabilities (PIP) when using the FUN2 panel (the reliabilities with the FUN2 panel were virtually identical to those with the FUN1 panel, but had the advantage of using fewer variants). We summed the PIP over 1 Mb windows to account for the possibility that different variants in LD might capture the same effect, and identified regions with a cumulative PIP > 0.5 (e.g., regions that had an additional effect in more than half of the iterations) (e.g., [53]). We identified 5 to 12 regions per trait, many of which overlapped with the 15 large effect variants fine-mapped by Gualdrón Duarte et al. [33].

For genomic selection using subsets of variants, we also compared the heritability estimates and dispersion bias (measured as the coefficient of regression of trait deviations on genomic predictions) obtained with the different models (see Additional file 2: Tables S11 and S12). The heritabilities estimated with BayesCπ and BSLMM were approximately 16% and 9% higher than those estimated with GBLUP-C, respectively, and should be treated with caution. BayesCπ also showed more dispersion bias than the other methods, whereas BSLMM generally showed less dispersion bias than GBLUP.

## Discussion

### Increased prediction accuracy with whole-genome sequence data

In this study, we used whole-genome sequence data, with or without annotation, to improve the accuracy of genomic prediction of muscular development traits in BBC. Contrary to some previous studies [4, 11, 12], the use of full-sequence data increased the reliability of breeding values compared to the use of MMD arrays. Several factors may explain this improvement, including more reliable genotype imputation due to ever-expanding reference panels and improving imputation software, and the benefits of ever-larger reference populations for genomic prediction. Although the benefit of sequence data was systematic, it remained relatively modest (e.g., + 1.8% reliability on average with BayesR) and was not always significant. This is consistent with our previous findings on simulated data [9], which showed that the use of whole-genome sequence data allowed to increase reliability mainly when rare variants contributed to genetic variance and were accurately genotyped or imputed. Conversely, little gain was obtained when common variants accounted for the largest proportion of genetic variance, as observed here. The segregation of common variants with large effects on muscular development traits in BBC or height has been previously described [33], while it remains difficult to study the importance of rare variants. Indeed, the imputation accuracy for rare variants remains low and we discarded variants with MAF < 0.01 or with low imputation accuracy. Therefore, we could not fully exploit the variation associated with rare variants.

### Relative performance of prediction models

Our study was also informative about the differences between methods. With full sequence data, BayesR and BayesRR-RC were the only Bayesian models to individually fit all the SNPs that could be run on our cluster, thanks to their implementation in the GMRM software. BayesR had higher reliabilities than GBLUP-S (+ 1.2% on average), while the differences were smaller when compared to GBLUP-C (+ 0.2% on average). Indeed, in our study, the use of centered genotypes consistently performed better than standardized genotypes (commonly used in human studies). This ranking is in agreement with previous studies that have been carried out in BBC [54] and in some other cattle breeds (e.g., [55]). In agreement, we also observed that selecting common variants (ARRAY panel) with methods using standardized genotypes and giving more weight to rare alleles (e.g., GBLUP-S) increased the reliability. The relationship between MAF and effect size has previously been linked to ongoing selection in the populations analyzed, with the architecture corresponding to standardized genotypes (i.e. rare variants having larger effects) being associated with purifying selection, whereas centered genotypes (i.e., common variants accounting for large proportions of genetic variance) are associated with directional selection [40]. Overall, these results therefore suggest directional selection for muscular development traits in BBC and support the use of centered genotypes. Bayesian models that allow for variants with large effects, such as BayesR, have been shown to have the potential to achieve higher prediction accuracies than GBLUP [17, 56–58]. In the present study, the advantage was rather in the lower bounds. Several elements could explain this observation. First, these models are expected to perform better than GBLUP especially when large effect variants contribute to the genetic architecture of target traits and less so for highly polygenic traits [41, 56, 58–60]. In addition, as sample size increases, SNP-BLUP also captures the large effect variants better (e.g. [61]). We must also bear in mind that estimating the effects of more than 10 million variants simultaneously with 14K genotyped individuals remains a challenging task and may require further iterations. Importantly, the reliability of BayesRR-RC could be further increased when using centered genotypes in livestock species. When using a subset of SNPs, the BSLMM approach [41] was consistently the best of all approaches, suggesting that a model combining a polygenic model with a few variants with an additional effect is efficient. We also tested two other similar models fitting a polygenic term and a group of large effect variants, Bolt-LMM [62] and BayesGC [63], but these did not perform as well as BSLMM (data not shown). In fact, such approaches could also be fitted with the BayesR framework, as the number of mixtures and their relative variance can be modified. Note also that BayesR and various extensions, including BayesRC [18] or BayesRCO [19], have been implemented in different programs [19, 57, 58] and that some of these may achieve higher accuracy, due to differences in model assumptions, settings or genotype coding (centered versus standardized genotypes). For example, we previously observed smaller differences between BSLMM and BayesR on a smaller data set with fewer markers, although BSLMM was still more accurate on average [54].

### Benefits of using functional annotation

This study is one of the first to use full sequence data to perform genomic prediction using models that incorporate functional information from experimental data in livestock species and using a relatively large cohort of individuals. For Bayesian mixture models, this was possible thanks to the development of software such as GMRM. However, this approach did not result in the strong improvement in reliability reported for humans [20, 64, 65]. This may be due to differences in population structure and past selection history (see above). In livestock species, effective population size is generally lower (e.g., [66, 67]), while LD extends at longer range (e.g., [68]) and relatedness is higher, as evidenced by the level of inbreeding observed in BBC [69]. This makes it more difficult to disentangle contributions from different functional classes, which are more confounded, as shown in the present study or previously in Yuan et al. [34]. As a result, the estimated enrichment levels are imprecise and the functional annotation is less optimally used. Another major difference is that the sample sizes used in human studies are much larger, providing more information to estimate different parameters. The genetic architecture also differs, as we have previously observed. While coding variants with large effects are rare and common variants have small effects and are generally regulatory in complex traits studied in humans [40, 70, 71], common coding variants with large effects are regularly observed in livestock species [32, 33, 56]. In addition, rare variants remain more difficult to exploit in livestock species due to low imputation accuracies and smaller reference panels. Finally, the amount and quality of functional information available in human studies is still much higher than in livestock species, allowing more genomic features to be fitted, such as in the so-called LD-baseline model including up to 53 groups. Better functional annotation could be achieved for the BBC breed by generating breed-specific regulatory variant catalogues using large numbers of individuals. Finally, additional categories could be considered, such as conservation scores, which have been shown to be relevant in both humans and livestock [28, 72].

When analyzing muscular development traits in BBC, the parameters estimated by models incorporating functional information were highly variable across traits and methods, and sometimes difficult to interpret biologically (e.g., null variance associated with some categories). These parameters should thus be interpreted with caution [34], especially for small categories such as coding variants or eQTLs. In addition, the estimates are highly dependent on the definition of the different functional categories, which may differ between studies, making comparisons difficult. Although the enrichment levels of the different categories are ranked as expected, we do not recommend evaluating their absolute values.

In such settings, the MGFBLUP framework produced slightly higher accuracies than GBLUP, probably because these models had greater flexibility, whereas BayesRR-RC tended to decrease accuracy compared to BayesR. Even without annotation, this Bayesian approach already has great flexibility (i.e., allowing some variants to have larger effects), and there may be less benefit in adding more flexibility, especially when more parameters need to be estimated and the reference population is not large enough. We have observed the difficulties and challenges of estimating all these parameters and individual variant effect simultaneously with BayesRR-RC. A potential disadvantage of BayesRR-RC is that when annotation is used, the variance used to model the SNP effects is reduced. For example, without annotation, the largest effects of coding variants are sampled from a distribution corresponding to 1% of the total genetic variance, whereas with annotation this becomes 1% of the variance explained by coding variants (i.e., if coding variants account for 10% of the SNP heritability, the effects are sampled from a distribution with a 10 times lower variance). This problem can be addressed in BayesRR-RC by fine-tuning the parameters of the model, as done by Orliac et al. [23]. Note also that the BayesRC model [18] uses the total genetic variance to model the SNP effects, which makes the model more robust to this problem and has the advantage of reducing the number of parameters to be estimated. Overall, in our setting, the accuracy of the models seems to result from their ability to capture the polygenic terms and the large effect variants, even with non-causal markers in LD with the causative variants, rather than from their ability to exploit the functional information.

### Future directions

Using BSLMM on large subsets of variants, selected based on their functional annotation or LD pruning, gave the highest reliabilities and significantly improved genomic predictions. With BSLMM, about 50 to 100 variants with additional effects were fitted in each iteration. Ideally, we should identify these 50–100 variants, or eventually a few more, and fit a model with a polygenic term and only these additional variants. It remains difficult to identify these variants with simple functional annotation because many of the functional classes are too large and lack specificity. For example, the number of coding variants and eQTLs is much larger (even if we target the 1000 variants with the largest contribution to genetic variance), although they do not include all the large effect variants. Improved fine-mapping approaches using functional annotation are needed to identify more of these variants, as in general only a handful of causative variants are currently unambiguously identified. Further improvements in functional annotation are therefore needed, including experimental data in the most relevant tissues, experiments on relatively large samples of individuals from the same breeds, and finer annotation levels. For example, by defining categories that combine motifs of transcription factor that are specific to the correct pathway and their levels of conservation. It would also be important to be able to identify which synonymous variants, assumed to be neutral, have an effect on the traits of interest. Finally, additional work is required to better exploit rare variants, for which imputation accuracy remains low, in genomic prediction.

## Conclusions

Compared to the GBLUP approach using medium maker density, as in the current genomic evaluation, the use of imputed whole-genome sequence data allowed to increase the reliability of genomic predictions for muscular development traits in BBC (+ 1.8% on average with the best method). Selection of subsets of markers based on functional annotation or LD pruning, allowed equivalent accuracy to be achieved at lower computational cost, allowing more methods to be applied. Overall, a strategy using a large panel of pre-selected functional variants, including coding variants, eQTLs and variants in regulatory elements, with a Bayesian model fitting a polygenic term combined with fewer than 200 additional effect variants achieved the highest accuracies (+ 4.2% on average). Therefore, fine-mapping of these additional effect variants may prove effective in improving genomic prediction accuracy. Models directly incorporating functional annotation only slightly improved reliability at best. This suggests that better annotation categories should be used than in the present study, and that further efforts are needed to improve functional annotation in BBC. In addition, more work is needed to exploit the genetic variance associated with rare variants, which remain difficult to impute accurately.

## Supplementary Information

Additional file 1: Table S1. Number of individuals and markers per genotyping array. Table S2. Estimated %SNP heritability (proportion of genetic variance explained by a category) and enrichment levels (relative variant effect size per category) for different functional categories with two annotation models, FAN1 and FAN2 when applied to shoulder muscling. Table S3. Estimated %SNP heritability (proportion of genetic variance explained by a category) and enrichment levels (relative variant effect size per category) for different functional categories with two annotation models, FAN1 and FAN2 when applied to top muscling. Table S4. Estimated %SNP heritability (proportion of genetic variance explained by a category) and enrichment levels (relative variant effect size per category) for different functional categories with two annotation models, FAN1 and FAN2 when applied to buttock muscling (side view). Table S5. Estimated %SNP heritability (proportion of genetic variance explained by a category) and enrichment levels (relative variant effect size per category) for different functional categories with two annotation models, FAN1 and FAN2 when applied to buttock muscling (rear view). Table S6. Estimated %SNP heritability (proportion of genetic variance explained by a category) and enrichment levels (relative variant effect size per category) for different functional categories with two annotation models, FAN1 and FAN2 when applied to global muscling score. Table S7. Gain of reliability achieved with BSLMM when using the FUN1 and LD99 panels.

Additional file 2: Table S8. Reliability obtained using whole-genome sequence data, with or without annotation, compared to that obtained using a medium marker density panel. Table S9. Reliability of different methods when using different marker panels selected on the basis of functional information or with an LD pruning strategy. Table S10. Reliability obtained using marker panels selected on the basis of functional information and for models with or without the incorporation of functional annotation. Table S11. Heritability estimated with different models when using different marker panels. Table S12. Bias of different models using different marker panels, measured as the coefficient of regression of trait deviations on genomic predictions.

Additional file 3: Figure S1. Significance levels of difference in reliabilities obtained with methods using whole-genome sequence data, with or without annotation. Figure S2. Significance levels of the difference in reliabilities obtained for each method when using subsets of markers selected based on LD pruning with different thresholds. Figure S3. Significance levels of the difference in reliabilities obtained between different methods when using a subset of markers selected based on LD pruning with of threshold at r^2^ > 0.99. Figure S4. Significance levels of the difference in reliability obtained for each method when using subsets of markers selected based on functional annotation, LD pruning or their presence on commercial arrays.

## Data Availability

The data sets used in the current study are the property of the Walloon Breeders Association and are not publicly available.
