# Supplementary material for: Evaluation of genomic selection models using whole genome sequence data and functional annotation in Belgian Blue cattle
Source: Genet Sel Evol. 2025 Mar 4;57:10. doi: 10.1186/s12711-025-00955-5 (PMC11881496; doi:10.1186/s12711-025-00955-5)
Supplement: Supplementary file 1 — Additional file 1: Table S1. Number of individuals and markers per genotyping array. Table S2. Estimated %SNP heritability (proportion of genetic variance explained by a category) and enrichment levels (relative variant effect size per category) for different functional categories with two annotation models, FAN1 and FAN2 when applied to shoulder muscling. Table S3. Estimated %SNP heritability (proportion of genetic variance explained by a category) and enrichment levels (relative variant effect size per category) for different functional categories with two annotation models, FAN1 and FAN2 when applied to top muscling. Table S4. Estimated %SNP heritability (proportion of genetic variance explained by a category) and enrichment levels (relative variant effect size per category) for different functional categories with two annotation models, FAN1 and FAN2 when applied to buttock muscling (side view). Table S5. Estimated %SNP heritability (proportion of genetic variance explained by a category) and enrichment levels (relative variant effect size per category) for different functional categories with two annotation models, FAN1 and FAN2 when applied to buttock muscling (rear view). Table S6. Estimated %SNP heritability (proportion of genetic variance explained by a category) and enrichment levels (relative variant effect size per category) for different functional categories with two annotation models, FAN1 and FAN2 when applied to global muscling score. Table S7. Gain of reliability achieved with BSLMM when using the FUN1 and LD99 panels. [file 12711_2025_955_MOESM1_ESM.docx]

Table S1. Number of individuals and markers per genotyping array.

| Genotyping array | Number of individuals | Number of variants | | Marker density |
| --- | --- | --- | --- | --- |
| LMD1 | 2467 | 9077 | Low | |
| LMD2 | 570 | 12,023 | Low | |
| LMD3 | 1511 | 12,638 | Low | |
| LMD4 | 8165 | 15,749 | Low | |
| LMD5 | 583 | 16,381 | Low | |
| MMD1 | 4930 | 48,699 | Medium | |
| MMD2 | 13,036 | 54,748 | Medium | |
| MMD3 | 1843 | 62,227 | Medium | |
| MMD4 | 1690 | 68,454 | Medium | |
| MMD5 | 1394 | 49,229 | Medium | |

Table S2. Estimated %SNP heritability (proportion of genetic variance explained by a category) and enrichment levels (relative variant effect size per category) for different functional categories with two annotation models, FAN1 and FAN2, when applied to shoulder muscling. MGFBLUP models were applied with GRMs constructed with either centered or standardized genotypes, respectively. The annotation categories are described in the Methods section.

|  | Annotation Group  (compartment where variants are located) | %SNP heritability | | | Enrichment | | | |  |
| --- | --- | --- | --- | --- | --- | --- | --- | --- | --- |
|  |  | MGFBLUP  Centered | MGFBLUP  Standardized | BayesRR-RC | | MGFBLUP  Centered | MGFBLUP  Standardized | BayesRR-RC | |
| FAN1 | Coding variants | 2.36 | 5.06 | 0.01 | | 6.35 | 13.63 | 0.03 | |
|  | eQTLs | 17.53 | 20.07 | 5.76 | | 82.88 | 94.85 | 27.25 | |
|  | Regulatory elements detected by ATAC-SEQ | 23.19 | 17.18 | 10.36 | | 3.06 | 2.26 | 1.36 | |
|  | Regulatory elements detected with epigenetic data | 11.41 | 0.02 | 12.81 | | 2.98 | 0.00 | 3.34 | |
|  | Regulatory elements detected with both techniques | 0.79 | 7.17 | 14.19 | | 0.27 | 2.42 | 4.78 | |
|  | Exon-associated elements | 0.06 | 0.04 | 9.10 | | 0.01 | 0.01 | 1.40 | |
|  | Intronic regions | 28.54 | 34.47 | 24.14 | | 1.07 | 1.30 | 0.91 | |
|  | Intergenic regions | 16.12 | 15.99 | 23.63 | | 0.31 | 0.31 | 0.45 | |
| FAN2 | Coding variants | 1.28 | 3.98 | 0.00 | | 3.46 | 10.73 | 0.00 | |
|  | eQTLs detected in muscle | 7.07 | 8.01 | 0.69 | | 167.43 | 189.87 | 16.36 | |
|  | eQTLs detected in other tissues | 13.45 | 14.13 | 10.70 | | 69.03 | 72.52 | 54.92 | |
|  | Regulatory elements detected in muscle | 8.63 | 11.25 | 14.17 | | 12.09 | 15.77 | 19.86 | |
|  | Regulatory elements detected in other tissues | 18.94 | 7.11 | 21.74 | | 1.39 | 0.52 | 1.59 | |
|  | Exon-associated elements | 0.07 | 0.08 | 13.97 | | 0.01 | 0.01 | 2.15 | |
|  | Intronic regions | 30.35 | 35.51 | 21.11 | | 1.14 | 1.34 | 0.79 | |
|  | Intergenic regions | 20.21 | 19.93 | 17.63 | | 0.39 | 0.38 | 0.34 | |

Table S3. Estimated %SNP heritability (proportion of genetic variance explained by a category) and enrichment levels (relative variant effect size per category) for different functional categories with two annotation models, FAN1 and FAN2, when applied to top muscling. MGFBLUP models were applied with GRMs constructed with either centered or standardized genotypes, respectively. The annotation categories are described in the Methods section.

|  | Annotation Group  (compartment where variants are located) | %SNP heritability | | | Enrichment | | | |  |
| --- | --- | --- | --- | --- | --- | --- | --- | --- | --- |
|  |  | MGFBLUP  Centered | MGFBLUP  Standardized | BayesRR-RC | | MGFBLUP  Centered | MGFBLUP  Standardized | BayesRR-RC | |
| FAN1 | Coding variants | 10.88 | 13.57 | 16.76 | | 29.32 | 36.58 | 45.17 | |
|  | eQTLs | 15.81 | 18.13 | 1.58 | | 74.71 | 85.69 | 7.46 | |
|  | Regulatory elements detected by ATAC-SEQ | 21.32 | 16.68 | 9.27 | | 2.81 | 2.20 | 1.22 | |
|  | Regulatory elements detected with epigenetic data | 14.29 | 11.87 | 8.70 | | 3.73 | 3.10 | 2.27 | |
|  | Regulatory elements detected with both techniques | 1.91 | 0.00 | 12.91 | | 0.64 | 0.00 | 4.35 | |
|  | Exon-associated elements | 0.62 | 0.17 | 7.38 | | 0.10 | 0.03 | 1.13 | |
|  | Intronic regions | 24.00 | 26.88 | 19.00 | | 0.90 | 1.01 | 0.72 | |
|  | Intergenic regions | 11.17 | 12.70 | 24.41 | | 0.21 | 0.24 | 0.47 | |
| FAN2 | Coding variants | 11.07 | 14.16 | 1.25 | | 29.83 | 38.16 | 3.37 | |
|  | eQTLs detected in muscle | 1.84 | 4.75 | 0.02 | | 43.58 | 112.57 | 0.44 | |
|  | eQTLs detected in other tissues | 13.56 | 13.38 | 5.01 | | 69.61 | 68.65 | 25.74 | |
|  | Regulatory elements detected in muscle | 14.13 | 14.68 | 19.19 | | 19.81 | 20.59 | 26.90 | |
|  | Regulatory elements detected in other tissues | 19.09 | 8.94 | 4.36 | | 1.40 | 0.65 | 0.32 | |
|  | Exon-associated elements | 1.98 | 0.19 | 19.45 | | 0.30 | 0.03 | 2.99 | |
|  | Intronic regions | 25.00 | 27.94 | 24.69 | | 0.94 | 1.05 | 0.93 | |
|  | Intergenic regions | 13.32 | 15.96 | 26.02 | | 0.26 | 0.31 | 0.50 | |

Table S4. Estimated %SNP heritability (proportion of genetic variance explained by a category) and enrichment levels (relative variant effect size per category) for different functional categories with two annotation models, FAN1 and FAN2, when applied to buttock muscling (side view). MGFBLUP models were applied with GRMs constructed with either centered or standardized genotypes, respectively. The annotation categories are described in the Methods section.

|  | Annotation Group  (compartment where variants are located) | %SNP heritability | | | Enrichment | | | |  |
| --- | --- | --- | --- | --- | --- | --- | --- | --- | --- |
|  |  | MGFBLUP  Centered | MGFBLUP  Standardized | BayesRR-RC | | MGFBLUP  Centered | MGFBLUP  Standardized | BayesRR-RC | |
| FAN1 | Coding variants | 6.77 | 4.70 | 10.71 | | 18.24 | 12.68 | 28.87 | |
|  | eQTLs | 13.13 | 15.28 | 2.73 | | 62.06 | 72.25 | 12.89 | |
|  | Regulatory elements detected by ATAC-SEQ | 24.32 | 19.43 | 19.15 | | 3.21 | 2.56 | 2.52 | |
|  | Regulatory elements detected with epigenetic data | 5.75 | 10.35 | 15.65 | | 1.50 | 2.70 | 4.08 | |
|  | Regulatory elements detected with both techniques | 13.11 | 10.42 | 0.76 | | 4.42 | 3.51 | 0.26 | |
|  | Exon-associated elements | 0.02 | 0.02 | 6.82 | | 0.00 | 0.00 | 1.05 | |
|  | Intronic regions | 18.78 | 21.89 | 20.28 | | 0.71 | 0.82 | 0.76 | |
|  | Intergenic regions | 18.11 | 17.90 | 23.89 | | 0.35 | 0.34 | 0.46 | |
| FAN2 | Coding variants | 7.34 | 5.79 | 0.01 | | 19.77 | 15.61 | 0.03 | |
|  | eQTLs detected in muscle | 2.21 | 0.03 | 0.26 | | 52.39 | 0.80 | 6.05 | |
|  | eQTLs detected in other tissues | 10.07 | 12.86 | 6.34 | | 51.67 | 65.99 | 32.56 | |
|  | Regulatory elements detected in muscle | 8.21 | 8.09 | 15.78 | | 11.51 | 11.34 | 22.13 | |
|  | Regulatory elements detected in other tissues | 32.72 | 34.55 | 26.01 | | 2.39 | 2.53 | 1.90 | |
|  | Exon-associated elements | 0.02 | 0.03 | 11.40 | | 0.00 | 0.00 | 1.75 | |
|  | Intronic regions | 19.49 | 20.95 | 16.36 | | 0.73 | 0.79 | 0.62 | |
|  | Intergenic regions | 19.94 | 17.70 | 23.85 | | 0.38 | 0.34 | 0.46 | |

Table S5. Estimated %SNP heritability (proportion of genetic variance explained by a category) and enrichment levels (relative variant effect size per category) for different functional categories with two annotation models, FAN1 and FAN2, when applied to buttock muscling (rear view). MGFBLUP models were applied with GRMs constructed with either centered or standardized genotypes, respectively. The annotation categories are described in the Methods section.

|  | Annotation Group  (compartment where variants are located) | %SNP heritability | | | Enrichment | | | |  |
| --- | --- | --- | --- | --- | --- | --- | --- | --- | --- |
|  |  | MGFBLUP  Centered | MGFBLUP  Standardized | BayesRR-RC | | MGFBLUP  Centered | MGFBLUP  Standardized | BayesRR-RC | |
| FAN1 | Coding variants | 1.86 | 2.80 | 11.84 | | 5.02 | 7.54 | 31.91 | |
|  | eQTLs | 15.32 | 19.30 | 3.05 | | 72.43 | 91.23 | 14.43 | |
|  | Regulatory elements detected by ATAC-SEQ | 14.09 | 2.60 | 13.69 | | 1.86 | 0.34 | 1.80 | |
|  | Regulatory elements detected with epigenetic data | 10.97 | 12.17 | 10.72 | | 2.86 | 3.17 | 2.80 | |
|  | Regulatory elements detected with both techniques | 2.06 | 0.00 | 14.40 | | 0.70 | 0.00 | 4.85 | |
|  | Exon-associated elements | 0.32 | 0.08 | 0.01 | | 0.05 | 0.01 | 0.00 | |
|  | Intronic regions | 38.87 | 43.94 | 24.29 | | 1.46 | 1.65 | 0.91 | |
|  | Intergenic regions | 16.50 | 19.11 | 21.99 | | 0.32 | 0.37 | 0.42 | |
| FAN2 | Coding variants | 1.67 | 3.15 | 0.00 | | 4.51 | 8.48 | 0.00 | |
|  | eQTLs detected in muscle | 6.08 | 4.82 | 0.00 | | 144.01 | 114.10 | 0.06 | |
|  | eQTLs detected in other tissues | 9.86 | 14.61 | 3.27 | | 50.60 | 75.01 | 16.77 | |
|  | Regulatory elements detected in muscle | 4.77 | 1.33 | 12.38 | | 6.69 | 1.86 | 17.35 | |
|  | Regulatory elements detected in other tissues | 21.75 | 16.83 | 27.21 | | 1.59 | 1.23 | 1.99 | |
|  | Exon-associated elements | 0.31 | 0.16 | 4.11 | | 0.05 | 0.03 | 0.63 | |
|  | Intronic regions | 38.39 | 41.86 | 28.25 | | 1.45 | 1.58 | 1.06 | |
|  | Intergenic regions | 17.17 | 17.24 | 24.78 | | 0.33 | 0.33 | 0.48 | |

Table S6. Estimated %SNP heritability (proportion of genetic variance explained by a category) and enrichment levels (relative variant effect size per category) for different functional categories with two annotation models, FAN1 and FAN2, when applied to global muscling score. MGFBLUP models were applied with GRMs constructed with either centered or standardized genotypes, respectively. The annotation categories are described in the Methods section.

|  | Annotation Group  (compartment where variants are located) | %SNP heritability | | | Enrichment | | | |  |
| --- | --- | --- | --- | --- | --- | --- | --- | --- | --- |
|  |  | MGFBLUP  Centered | MGFBLUP  Standardized | BayesRR-RC | | MGFBLUP  Centered | MGFBLUP  Standardized | BayesRR-RC | |
| FAN1 | Coding variants | 8.01 | 12.11 | 0.63 | | 21.59 | 32.62 | 1.70 | |
|  | eQTLs | 12.78 | 13.88 | 8.65 | | 60.43 | 65.63 | 40.91 | |
|  | Regulatory elements detected by ATAC-SEQ | 19.47 | 8.91 | 13.21 | | 2.57 | 1.17 | 1.74 | |
|  | Regulatory elements detected with epigenetic data | 1.29 | 0.04 | 10.83 | | 0.34 | 0.01 | 2.82 | |
|  | Regulatory elements detected with both techniques | 21.45 | 23.48 | 15.71 | | 7.23 | 7.91 | 5.29 | |
|  | Exon-associated elements | 0.06 | 0.03 | 12.14 | | 0.01 | 0.01 | 1.87 | |
|  | Intronic regions | 24.15 | 28.36 | 20.29 | | 0.91 | 1.07 | 0.76 | |
|  | Intergenic regions | 12.78 | 13.18 | 18.54 | | 0.25 | 0.25 | 0.36 | |
| FAN2 | Coding variants | 8.02 | 13.08 | 15.97 | | 21.62 | 35.23 | 43.03 | |
|  | eQTLs detected in muscle | 5.64 | 5.93 | 0.24 | | 133.68 | 140.44 | 5.64 | |
|  | eQTLs detected in other tissues | 9.46 | 8.60 | 3.76 | | 48.57 | 44.16 | 19.29 | |
|  | Regulatory elements detected in muscle | 11.00 | 12.25 | 6.94 | | 15.42 | 17.18 | 9.73 | |
|  | Regulatory elements detected in other tissues | 29.79 | 23.27 | 20.64 | | 2.18 | 1.70 | 1.51 | |
|  | Exon-associated elements | 0.06 | 0.04 | 12.38 | | 0.01 | 0.01 | 1.90 | |
|  | Intronic regions | 22.52 | 24.93 | 19.72 | | 0.85 | 0.94 | 0.74 | |
|  | Intergenic regions | 13.50 | 11.90 | 20.36 | | 0.26 | 0.23 | 0.39 | |

Table S7. Gain of reliability achieved with BSLMM when using panel FUN1 and LD99. Reliabilities were compared to those obtained with GBLUP using centered genotype and medium density array. Significance levels were estimated by 10,000 bootstraps.

| Traits | FUN1 | | LD99 | |
| --- | --- | --- | --- | --- |
|  | Gain of reliability | P-value | Gain of reliability | P-value |
| Top | 0.052 | <0.0001 | 0.043 | <0.0001 |
| Shoulder | 0.038 | 0.0026 | 0.031 | 0.0082 |
| Buttock (rear) | 0.038 | 0.0014 | 0.033 | 0.0048 |
| Buttock (side) | 0.036 | <0.0001 | 0.029 | 0.0008 |
| Global score | 0.049 | <0.0001 | 0.043 | <0.0001 |
