# Supplementary material for: Evaluation of genomic selection models using whole genome sequence data and functional annotation in Belgian Blue cattle
Source: Genet Sel Evol. 2025 Mar 4;57:10. doi: 10.1186/s12711-025-00955-5 (PMC11881496; doi:10.1186/s12711-025-00955-5)
Supplement: Supplementary file 3 — Additional file 3: Figure S1. Significance levels of difference in reliabilities obtained with methods using whole-genome sequence data, with or without annotation. Figure S2. Significance levels of the difference in reliabilities obtained for each method when using subsets of markers selected based on LD pruning with different thresholds. Figure S3. Significance levels of the difference in reliabilities obtained between different methods when using a subset of markers selected based on LD pruning with of threshold at r2 > 0.99. Figure S4. Significance levels of the difference in reliability obtained for each method when using subsets of markers selected based on functional annotation, LD pruning or their presence on commercial arrays. [file 12711_2025_955_MOESM3_ESM.docx]

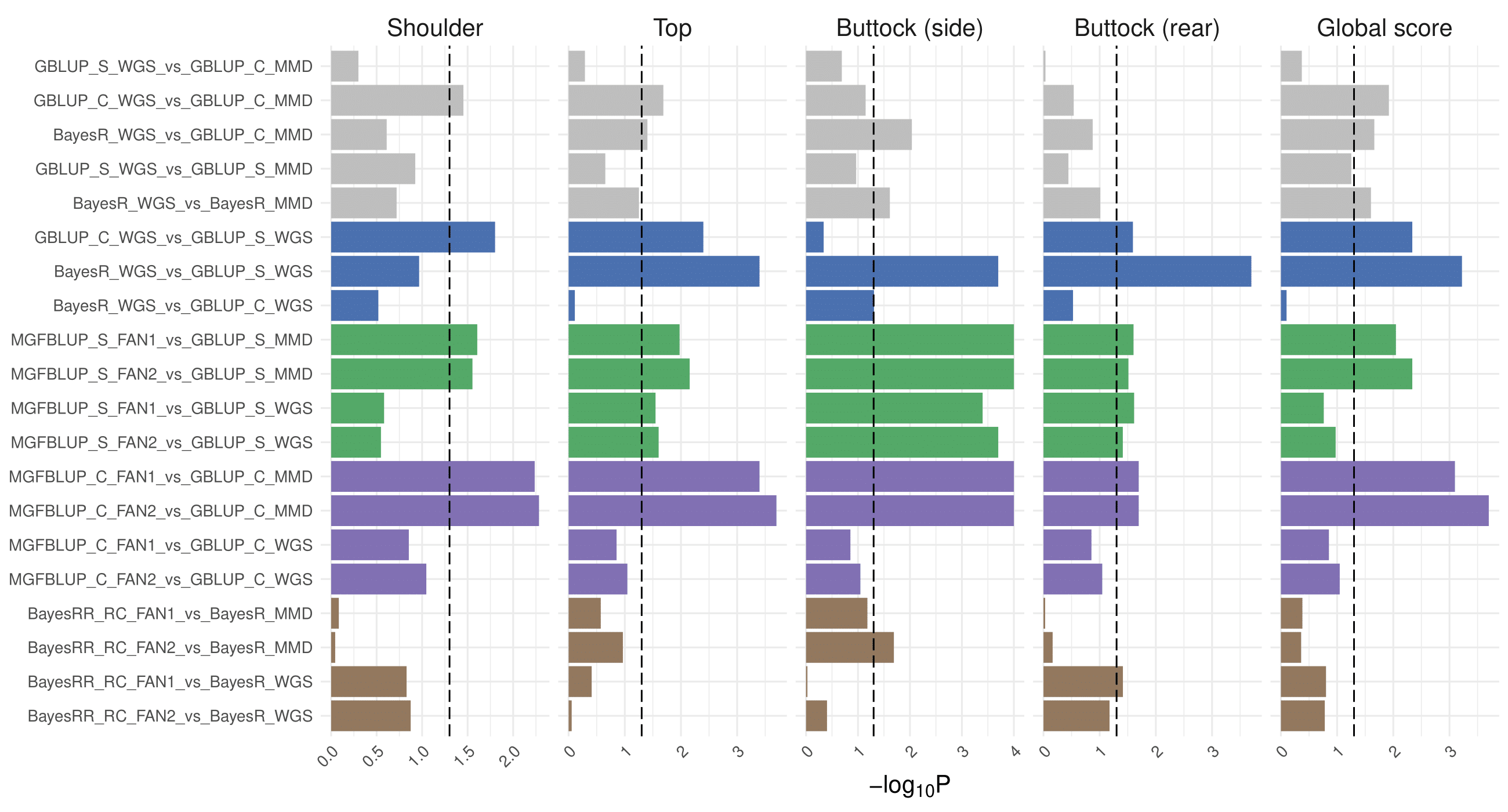


**Figure S1. Significance levels of difference in reliabilities obtained with methods using whole-genome sequence data, with or without annotation.** Comparisons were also made with medium marker density (MMD) array prediction. The names of the pair of methods compared are indicated on the left, with the extensions ‘-C’ and ‘-S’ indicating whether the GRMs used in GBLUP and MGFBLUP models were constructed with centered and standardized genotypes, respectively, WGS referring to the use of the whole-genome sequence data without annotation, FAN1 and FAN2 referring to the two models incorporating functional annotation (describing in Table 1). P-values of differences were obtained by bootstrapping and are presented on a -log10 scale, the dashed line indicates the significance threshold at p=0.05. The colors facilitate the reading of the results and indicate which pairs of methods are compared: gray for comparisons between WGS and MMD, blue for comparisons of the three methods at the sequence level, green for comparisons of GBLUP-S (without functional annotation) and MGFBLUP-S (with functional annotation) models, purple and brown for the same comparisons with GBLUP-C versus MGFBLUP-C models and with BayesR versus BayesRR-RC models, respectively.


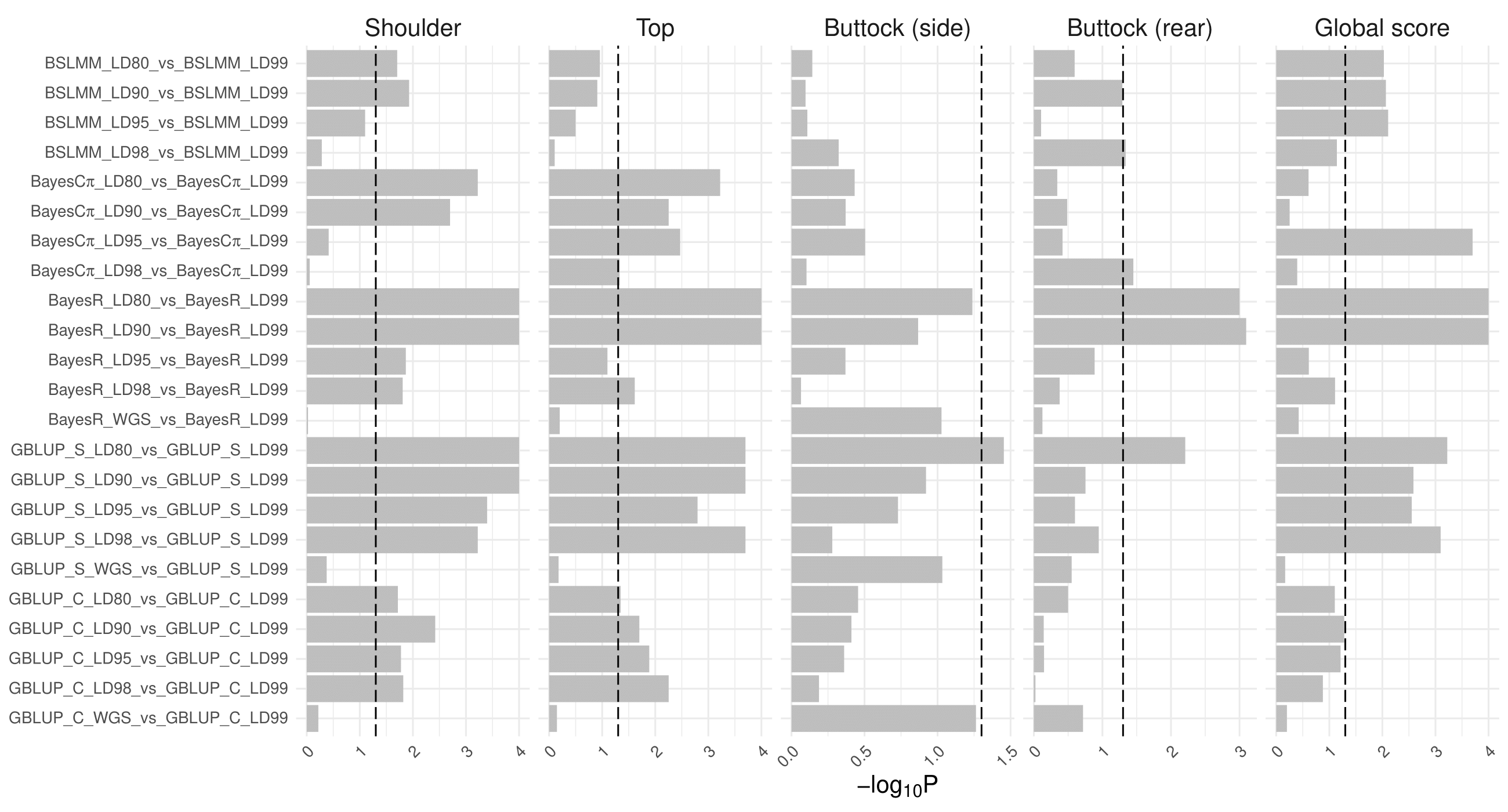


**Figure S2. Significance levels of the difference in reliabilities obtained for each method when using subsets of markers selected based on LD pruning with different thresholds.** The comparisons are made per method, with different selected subsets of markers. The names of the compared method pairs are given on the left, where GBLUP-S and GBLUP-C refer to GBLUP with centered and standardized genotypes, WGS refers to the use of whole-genome sequence data, and LD80 to LD99 refer to marker panels selected by LD pruning with the threshold set at r² > 80 to 99, respectively. P-values of differences were obtained by bootstrapping and are presented on a -log10 scale, the dashed line indicates the significance threshold at p=0.05.


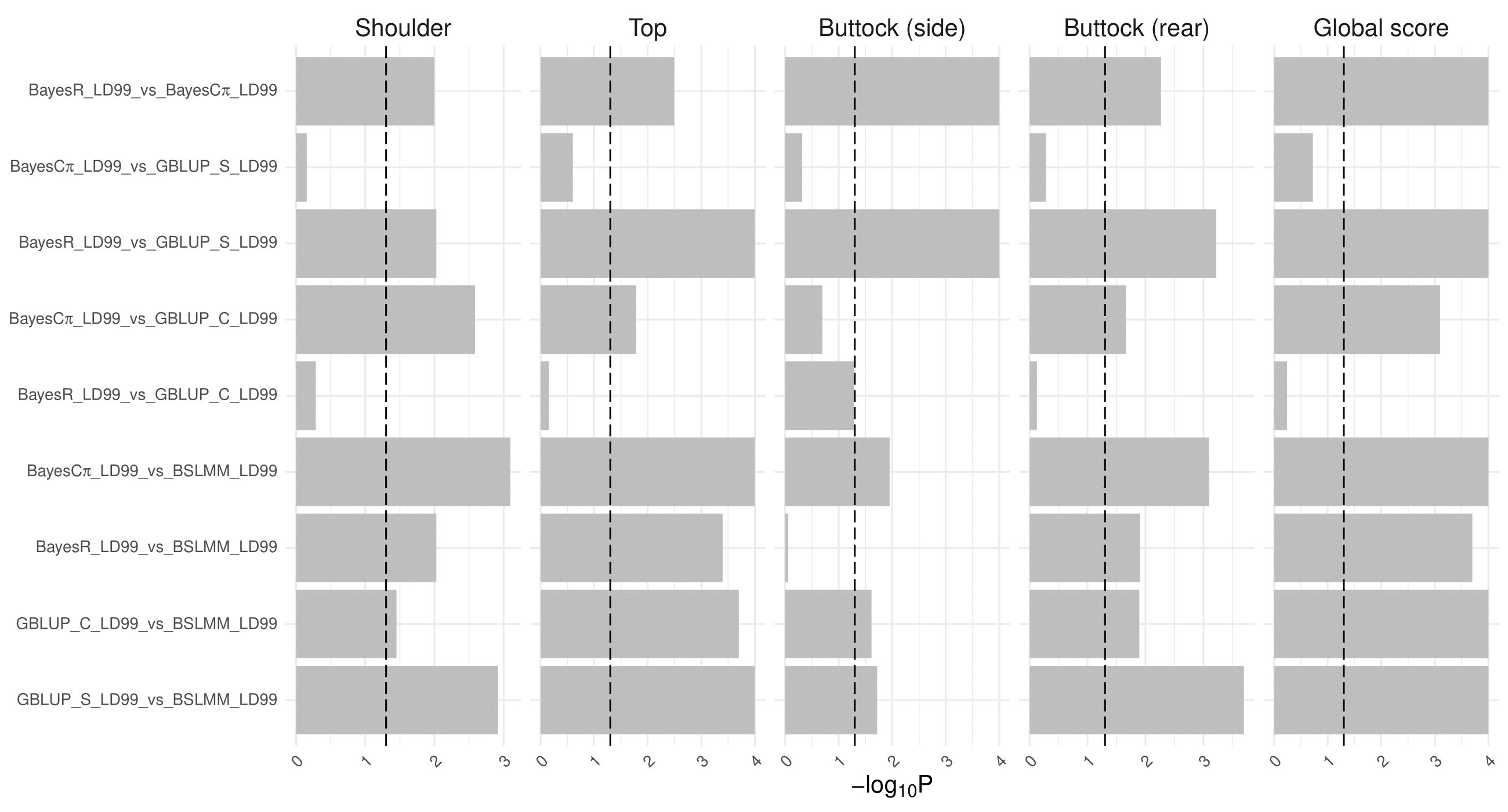


**Figure S3. Significance levels of the difference in reliabilities obtained between different methods when using a subset of markers selected based on LD pruning with of threshold at r² > 0.99.** The names of the compared method pairs are given on the left, where GBLUP-S and GBLUP-C refer to GBLUP with centered and standardized genotypes, LD99 refers to marker panels selected by LD pruning with the threshold set at r² > 99. P-values of differences were obtained by bootstrapping and are presented on a -log10 scale, the dashed line indicates the significance threshold at p=0.05.


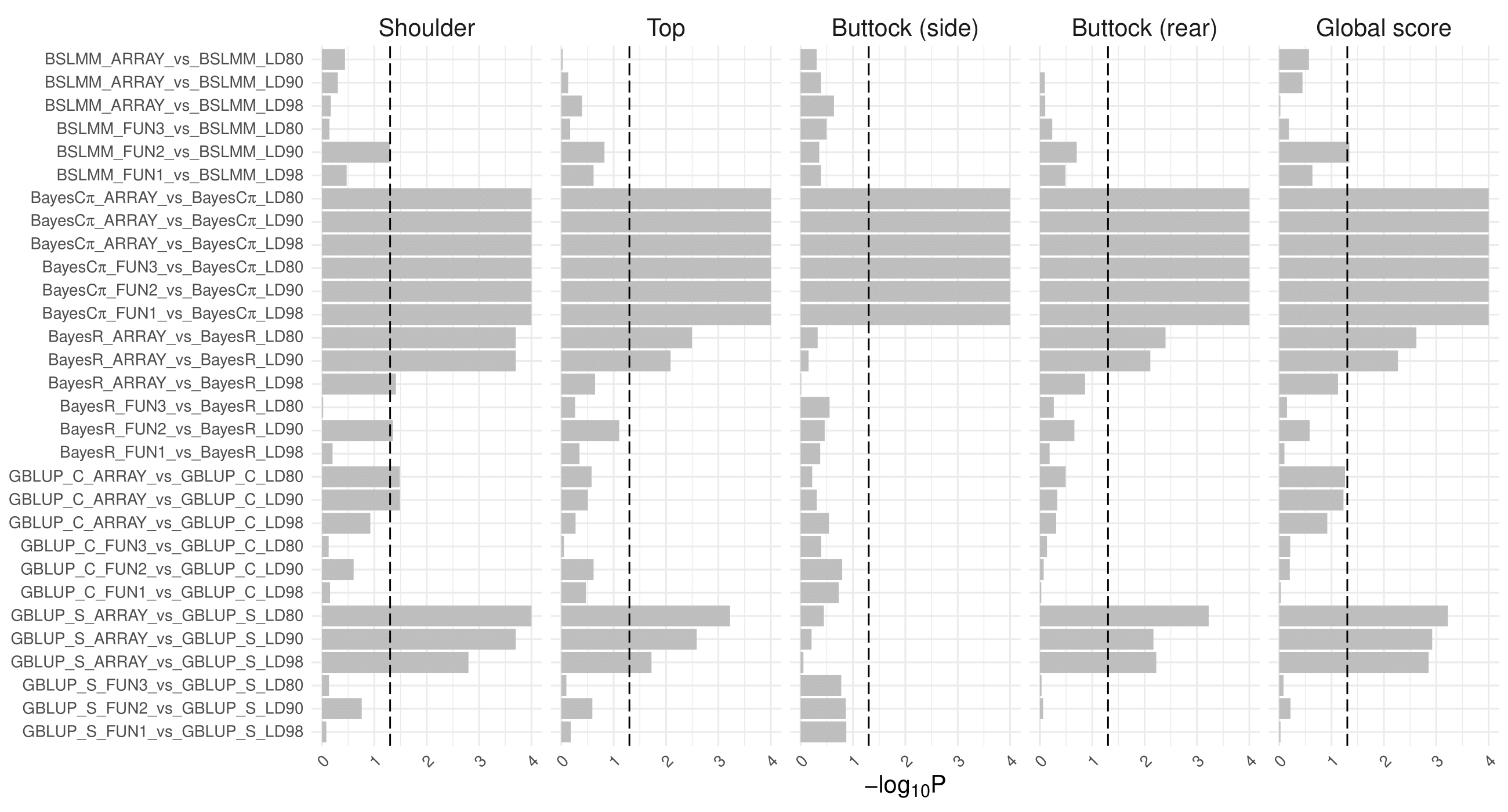


**Figure S4. Significance levels of the difference in reliability obtained for each method when using subsets of markers selected based on functional annotation, LD pruning or their presence on commercial arrays.** The comparisons are made per method, with different selected subsets of markers. The names of the compared method pairs are given on the left, where the extensions ‘-C’ and ‘-S’ indicate whether the GRM used in that GBLUP model was constructed with centered and standardized genotypes, respectively, FUN1 to FUN3 refer to marker panels selected based on functional annotation, ARRAY refers to markers present on commercial genotyping arrays, and LD80, LD90, and LD98 refer to marker panels selected by LD pruning with the threshold set at r² > 80, 90, and 98, respectively (see Table 2 for more details on the marker panels). FUN and LD panels were compared for panels of approximately the same size, with the number of markers on the ARRAY panel being approximately the same as on the FUN3 and LD80 panels. P-values of differences were obtained by bootstrapping and are presented on a -log10 scale, the dashed line indicates the significance threshold at p=0.05.
